# Risk of acute epiglottitis in patients with preexisting diabetes mellitus: A population-based case–control study

**DOI:** 10.1371/journal.pone.0199036

**Published:** 2018-06-11

**Authors:** Yao-Te Tsai, Ethan I. Huang, Geng-He Chang, Ming-Shao Tsai, Cheng-Ming Hsu, Yao-Hsu Yang, Meng-Hung Lin, Chia-Yen Liu, Hsueh-Yu Li

**Affiliations:** 1 Department of Otorhinolaryngology-Head and Neck Surgery, Chang Gung Memorial Hospital, Chiayi, Taiwan; 2 College of Medicine, Chang Gung University, Taoyuan, Taiwan; 3 Department of Traditional Chinese Medicine, Chang Gung Memorial Hospital, Chiayi, Taiwan; 4 Institute of Occupational Medicine and Industrial Hygiene, National Taiwan University College of Public Health, Taipei, Taiwan; 5 School of Traditional Chinese Medicine, College of Medicine, Chang Gung University, Taoyuan, Taiwan; 6 Health Information and Epidemiology Laboratory, Chang Gung Memorial Hospital, Chiayi, Taiwan; 7 Department of Otolaryngology–Head and Neck Surgery, Linkou Chang Gung Memorial Hospital, Taoyuan, Taiwan; National Yang-Ming University, TAIWAN

## Abstract

**Objective:**

Studies have revealed that 3.5%–26.6% of patients with epiglottitis have comorbid diabetes mellitus (DM). However, whether preexisting DM is a risk factor for acute epiglottitis remains unclear. In this study, our aim was to explore the relationship between preexisting DM and acute epiglottitis in different age and sex groups by using population-based data in Taiwan.

**Methods:**

We analyzed data between January 2000 and December 2013 obtained from the Taiwan National Health Insurance Research Database. The case group consisted of 2,393 patients with acute epiglottitis. The control group comprised 9,572 individuals without epiglottitis, frequency matched by sex, age, urbanization level, and income. Underlying DM was retrospectively assessed in the cases and controls. Univariate and multivariate logistic regression analyses were used to investigate the associations between underlying DM and acute epiglottitis.

**Results:**

Of the 2,393 patients, 180 (7.5%) had preexisting DM, whereas only 530 (5.5%) of the 9,572 controls had preexisting DM. Multivariate logistic regression analyses indicated that preexisting DM was significantly associated with acute epiglottitis (adjusted odds ratio [aOR] = 1.42, 95% confidence interval [CI] = 1.15–1.75, *P* = 0.004). Subgroup analysis showed that the association between DM and epiglottitis remained significant for men (aOR = 1.57, 95% CI: 1.19–2.08, *p* = 0.002) but not for women. Age-stratified analysis revealed a significant association between DM and acute epiglottitis in patients aged 35–64 years. Use of anti-diabetic agents was not significantly associated with the development of acute epiglottitis among diabetic patients, including oral hypoglycemic agents (OHA) alone (aOR = 0.88, 95% CI = 0.53–1.46, *p* = 0.616), and OHA combined with insulin/ insulin alone (aOR = 1.30, 95% CI = 0.76–2.22, *p* = 0.339). The association between presence of diabetes complications and the occurrence of acute epiglottitis was also not significant among diabetic patients in this study setting (aOR = 0.86, 95% CI = 0.59–1.26, *p* = 0.439).

**Conclusions:**

The results of our large-scale population-based case–control study indicate that preexisting DM is one of the possible factors associated with the development of acute epiglottitis. Physicians should pay attention to the symptoms and signs of acute epiglottitis in DM patients, particularly in men aged 35–64 years.

## Introduction

Epiglottitis is the acute inflammation of the supraglottic region, including the epiglottis, arytenoids, and aryepiglottic folds. It is a true airway emergency, and without timely intervention, the supraglottic swelling may lead to life-threatening airway obstruction[1, 2] and severe complications such as sepsis,[3] meningitis,[4] necrotizing fasciitis, and mediastinitis.[5–8] The risk factors for epiglottitis include old age, the male sex, obesity, a preexisting epiglottic cyst, and an impaired host immune system.[9–11] Infected epiglottic cysts and impaired immunity have also been reported to increase the risk of recurrent episodes.[9, 10] The most common pathogens implicated in acute epiglottitis are bacteria such as type-b *Haemophilus influenzae*, alpha- and beta-hemolytic *streptococci*, *Staphylococcus aureus*, *Escherichia coli*, *Enterobacter*, *Klebsiella pneumoniae*, and other *H*. *influenzae species*.[12] Other reported causes include viral infections, fungal infections, trauma by a foreign body, inhalation burns, and chemical ingestion.[13] However, despite detailed investigation, a specific pathogen can be identified from blood or throat cultures in only 10%–25% of patients with epiglottitis.[14, 15] The incidence of pediatric epiglottitis dropped dramatically after routine use of the *H*. *influenzae* type-b (Hib) vaccine in childhood vaccination programs.[16–20] However, the incidence of acute epiglottitis in adults has been either increasing[2, 12, 16, 21] or remaining constant.[17, 22] Shah *et al*. conducted an 8-year retrospective review of epiglottitis admissions from 1998 to 2006 and concluded that epiglottitis continues to be a significant clinical entity in the United States and that the incidence of adult epiglottitis is increasing in two groups: those 45–64 years of age and those older than 85 years.[23] A common perception is that in the Hib vaccine era, acute epiglottitis has become a disease of adults and that the pathogens of epiglottitis have shifted to those other than Hib.[2, 24] A considerable number of adult patients with epiglottitis have preexisting medical conditions at diagnosis, such as diabetes mellitus (DM), hypertension, and alcohol abuse, which may weaken their immunity and increase their susceptibility to infections.[12, 15, 22, 25]

Studies has revealed that 3.5%–26.6% of patients with epiglottitis have comorbid DM,[26, 27] and some have life-threatening complications with a fulminant clinical course [28, 29]. Moreover, studies have indicated that the severity of epiglottitis is higher in patients with DM due to the higher 2-day mortality and the elevated risk of airway obstruction necessitating intervention in such patients than in those without DM.[9, 11, 21, 22, 30] Nevertheless, a quantitative relationship between DM and acute epiglottitis has not been established in pediatric or adult patients due to the disease rarity. In the present population-based study, our aim was to explore the relationship between preexisting DM and acute epiglottitis in different age and sex groups by using data from the National Health Insurance Research Database (NHIRD) in Taiwan.

## Material and methods

### Ethics statement

The study protocol was reviewed and approved by the Institutional Review Board (IRB) of Chang Gung Memorial Hospital (approval no. 201701635B1). Since the NHIRD contains only de-identified secondary data, the IRB waived the requirement of informed consent.

### Data source

The Taiwanese government implemented a compulsory National Health Insurance (NHI) program in March 1995, which is a nationwide health care system and provides medical services for the country’s 23.5 million residents. It covers over 99% of the population in Taiwan and records clinical diagnosis according to the International Classification of Diseases, Ninth Revision, Clinical Modification (ICD-9-CM) codes.[31] All claims data are collected in the NHIRD, which contains encrypted personal information and provides various medical data including records of registration, ambulatory and inpatient care, catastrophic illness, surgical procedure, and medication.

The data used in this study originated from the Longitudinal Health Insurance Database 2005 (LHID2005), which is a representative database of the NHIRD. The LHID2005 includes all the medical claims (1996–2013) of 1 million individuals randomly selected from the 2005 Registry of Beneficiaries of the NHIRD by using a systematic sampling method, representing approximately 5% of all people in Taiwan. According to the Taiwan National Health Research Institutes reports, no statistically significant differences exist in age, sex, or health care costs between the sample group and all enrollees in the LHID2005.[32]

### Study design and participants

**Fig 1:**
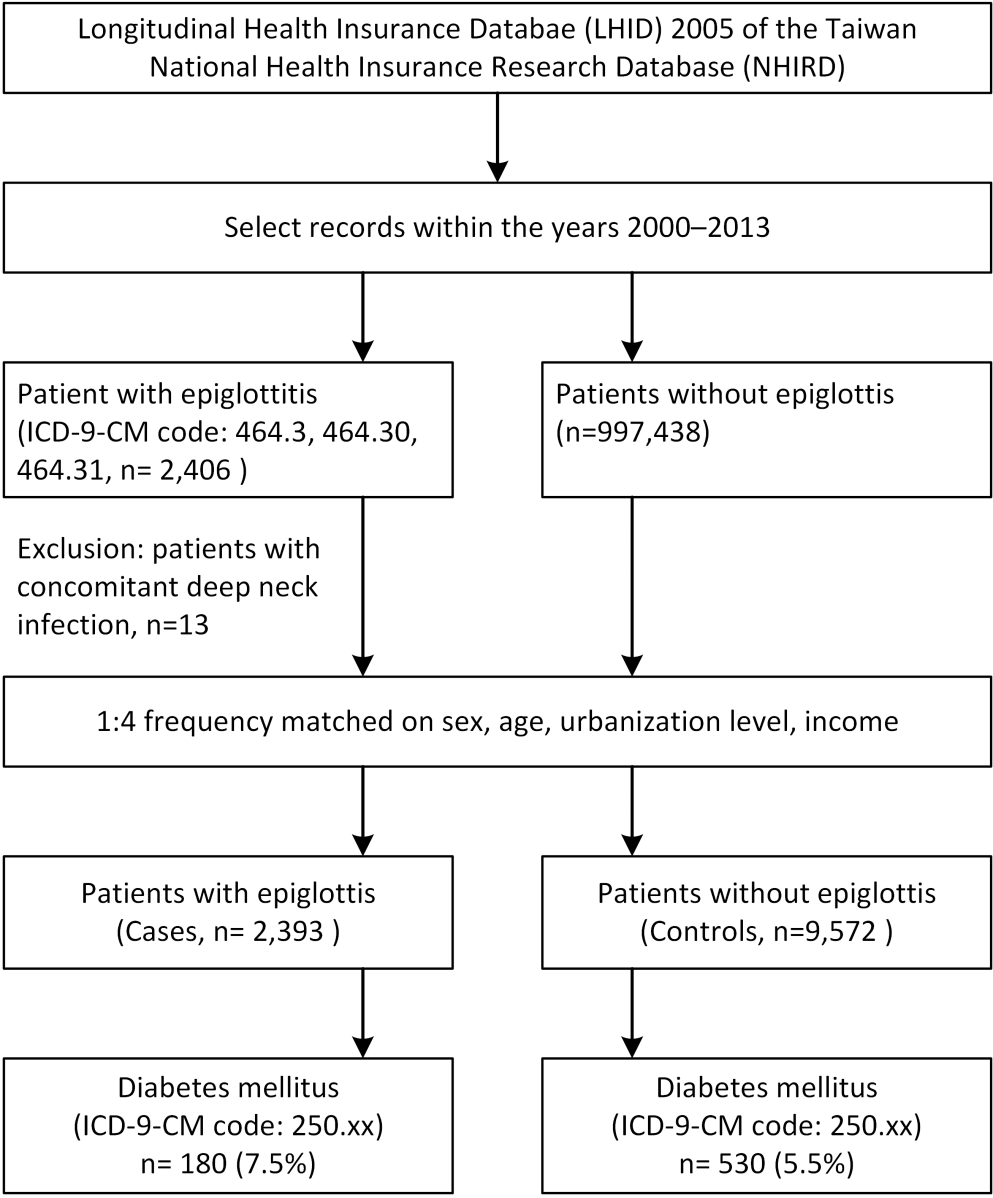
Flow diagram of the study. ICD-9-CM. International Classification of Diseases, Ninth Revision, Clinical Modification.

We categorized patients into an acute epiglottitis (case) group and a nonepiglottitis (control) group. Patients who met the following criteria were selected into the case group: (1) diagnosed as having acute epiglottitis based on the ICD-9-CM code 464.3, 464.30, or 464.31 by an otolaryngologist; (2) had two or more ambulatory visits or at least one inpatient visit for acute epiglottitis; and (3) had no concomitant deep neck infection—that is, ICD-9 code 528.3 (cellulitis and abscess of oral soft tissues), 478.22 (parapharyngeal abscess), 478.24 (retropharyngeal abscess), or 682.11 (cellulitis and abscess of neck) (Fig 1).[31] For each of these patients, the date of initial epiglottitis diagnosis was assigned as the index date. To increase statistical power, for each case identified on the index date, we randomly selected four individuals without acute epiglottitis as controls on the same day. Both cases and controls were identified from the LHID2005 with records between January 1, 2000, and December 31, 2013. The groups were frequency matched for sex, age, urbanization level, and income.

### Exposure assessment

DM in both cases and controls was assessed on the basis of at least three outpatient claims or at least one inpatient claim of ICD-9 Code 250.xx. Other medical conditions potentially associated with acute epiglottitis, including asthma (ICD-9-CM code: 493.xx), chronic liver disease and cirrhosis (ICD-9-CM code: 571.xx), coronary artery disease (ICD-9-CM codes: 410–414), hypertension (ICD-9-CM codes: 401–405), and pneumonia and influenza (ICD-9-CM codes: 480–488), upper digestive tract cancer (ICD-9-CM codes: 141–151), autoimmune diseases (ICD-9-CM codes: 714.0, 720, 720.0, 710.0, 370.33, 710.2, 710.1), chronic obstructive pulmonary disease (COPD, ICD-9-CM codes: 490–496), alcohol dependence and abuse (ICD-9-CM codes: 303, 303.xx, 305.0, 305.0x), corrosive injury of upper digestive tract (ICD-9-CM codes: 947.0–947.3), and gastroesophageal reflux disease (GERD, ICD-9-CM codes: 530.11, 530.81,530.85) were also assessed from the claims data[11, 13, 15, 26, 27, 33–37]. We included these comorbidities if they occurred either in the inpatient setting or in more than three ambulatory care claims. Comorbidities of each individual were all identified before the index date or matched index date, and each comorbidity was analyzed as a binomial variable.

In addition, adapted Diabetes Complications Severity Index (aDCSI) was computed to represent the presence of diabetes complications[38, 39]. The aDCSI includes following seven categories of diabetes complications: cardiovascular disease, nephropathy, retinopathy, peripheral vascular disease, cerebrovascular disease, neuropathy, and metabolic disease. Events were identified by using ICD-9-CM codes from both inpatient and outpatient records. Complications severity index was categorized into 2 or 3 levels (no abnormality = 0, some abnormality = 1, severe abnormality = 2), and neuropathy is the only complication with 2 levels (not present = 0, abnormal = 1). A total score of 0~13 was possible for the aDCSI score [40].

### Statistical analysis

The distributional properties of continuous variables are presented as mean and standard deviation, and categorical variables are presented as frequency and percentage. We evaluated the distributions of sex, age, urbanization level of patient’s residence, insured amount, and comorbidities between cases and controls by using the chi-squared test. The prevalence of diabetes was the main outcome of interest of this study. To reduce potential confounders, a logistic regression analysis was performed to evaluate the risk of epiglottitis associated with DM and various comorbidities (after adjustment for age, sex, urbanization level, insured amount, and comorbidities). All analyses were performed using SAS version 9.4 (SAS Institute, Cary, NC), and statistical significance was set at a two-sided *P* value of < 0.05.

## Results

Between 2000 and 2013, 2,393 newly coded patients with acute epiglottitis met the criteria for cases, and 9,572 individuals were matched as controls. Table 1 presents the intergroup demographic characteristics. No significant differences in sex, age, urbanization level, or income were observed between the groups because of frequency matching on these variables. The mean age for the total 11,965 patients was 33.6 years (standard deviation = 23.3 years). Half of the individuals were under 34 years old, and only 10.2% of the patients were over 65 years old. Among the 2,393 patients with acute epiglottitis, 180 (7.5%) had underlying DM, whereas 530 (5.5%) of the 9,572 controls had DM (*p* < .001). Most DM patients in both case and control groups were type 2 DM. Compared with the control group, the epiglottitis group had a higher incidence of asthma, chronic liver disease, coronary artery disease, and pneumonia and influenza, chronic obstructive pulmonary disease (COPD), autoimmune diseases, alcohol dependence and abuse, gastroesophageal reflux disease (GERD), and upper digestive tract cancer.

**Table 1:** Demographic characteristics and comorbid medical disorders of patients with epiglottitis and controls.

Variable	Epiglottitis (n = 2,393)		Controls (n = 9,572)		*P*-value
	n	%	n	%	
Sex					1.000
Men	1233	51.5	4932	51.5	
Women	1160	48.5	4640	48.5	
Age (years)					1.000
<34	1196	50.0	4784	50.0	
35–49	531	22.2	2124	22.2	
50–64	421	17.6	1684	17.6	
≥65	245	10.2	980	10.2	
Monthly income (NTD)					1.000
1–15840	312	13.0	1248	13.0	
15841–25000	593	24.8	2372	24.8	
≥25001	507	21.2	2028	21.2	
Urbanization level					1.000
1(City)	694	29.0	2776	29.0	
2	1099	45.9	4396	45.9	
3	491	20.5	1964	20.5	
4 (Village)	109	4.6	436	4.6	
Diabetes mellitus	180	7.5	530	5.5	<0.001
Type 1	4	0.3	14	0.2	0.263
Type 2	168	7.0	445	4.7	<0.001
Comorbidities					
Asthma	201	8.4	573	6.0	<0.001
Chronic liver disease	203	8.5	665	7.0	0.010
Coronary artery disease	174	7.3	580	6.1	0.029
Hypertension	343	14.3	1249	13.1	0.098
Pneumonia/ influenza	455	19.0	1225	12.8	<0.001
COPD	361	15.1	1058	11.1	<0.001
Alcohol dependence/abuse	12	0.5	23	0.2	0.034

Corrosive injury of digestive tract	2	0.1	4	0.04	-
GERD	63	2.6	130	1.4	<0.001
Autoimmune diseases	54	2.3	131	1.4	0.002
Upper digestive tract cancer	35	1.5	24	0.3	<0.001

Abbreviations: NTD, New Taiwan dollar. COPD indicates chronic obstructive pulmonary disease; GERD, gastroesophageal reflux disease.

Table 2 presents the results of the multivariate logistic regression analyses. After adjustment for age, sex, urbanization level, income, and comorbidities, results from the multivariable logistic regression analyses indicated that underlying DM was associated with acute epiglottitis (adjusted odds ratio [aOR] = 1.42, 95% confidence interval [CI] = 1.15–1.75, *p* = 0.004). Other comorbidities including pneumonia and influenza, COPD, autoimmune diseases, GERD, and upper digestive tract cancer also showed significant associations with acute epiglottitis. The subgroup analysis showed that the association between DM and epiglottitis remained significant for men (aOR = 1.57, 95% CI: 1.19–2.08, *p* = 0.002) but not for women (aOR = 1.23, 95% CI = 0.91–1.68, *p* = 0.181). Age-stratified analysis revealed remarkable associations between DM and epiglottitis among those aged 35–49 years (aOR = 2.12, 95% CI = 1.29–3.48, *p* = 0.003) and 50–64 years (aOR = 1.52, 95% CI = 1.10–2.09, *p* = 0.011).

**Table 2:** Multivariate logistic analyses of the association between acute epiglottitis and diabetes mellitus from 2000 to 2013 in Taiwan.

Variable	Adjusted OR*	95% CI	*p*-value
**Diabetes mellitus**			
No	1.00	Reference	
Yes	1.42	(1.15–1.75)	0.004
**Comorbidities**			
Asthma	0.96	(0.75–1.24)	0.769
Chronic liver disease	1.18	(0.98–1.41)	0.081
Coronary artery disease	1.04	(0.84–1.29)	0.715
Hypertension	1.13	(0.95–1.36)	0.172
Pneumonia and influenza	1.49	(1.31–1.69)	<0.001
COPD	1.31	(1.07–1.60)	0.008
Alcohol dependence/abuse	1.30	(0.62–2.74)	0.491
Corrosive injury of upper digestive tract	2.01	(0.36–11.14)	0.424
GERD	1.73	(1.26–2.38)	0.001
Autoimmune diseases	1.54	(1.10–2.14)	0.011
Upper digestive tract cancer	5.38	(3.17–9.12)	<0.001
**Subgroup effects**			
Sex			
Men	1.57	(1.19–2.08)	0.002
Women	1.23	(0.91–1.68)	0.181
Age (years)			
<34	0.50	(0.11–2.36)	0.379
35–49	2.12	(1.29–3.48)	0.003
50–64	1.52	(1.10–2.09)	0.011
≥65	1.25	(0.89–1.76)	0.192

Abbreviations: OR, odds ratio; CI, confidence interval

*The model was adjusted for sex, age, urbanization level, income, and comorbidities.

Table 3 lists the stratified analysis for the association between acute epiglottitis and DM with different definition of preexisting DM duration before the index date. After adjusting for the demographic factors and comorbidities, the association remained significant and constant when the underlying DM was diagnosed at least 3 months, 6 months, 1 year, and 3 years before the occurrence of acute epiglottitis (aOR = 1.28, 1.28, 1.27, 1.23; *p* = 0.023, 0.026, 0.032, and 0.035 respectively).

**Table 3:** Stratified analysis of the association between acute epiglottitis and DM with different definition of preexisting DM period before the index date.

DM period before index date	Crude OR (95% CI)	*p*-value	Adjusted OR* (95% CI)	*p*-value
≥ 3 months	1.44 (1.19–1.72)	<0.001	1.28 (1.04–1.58)	0.023
≥ 6 months	1.44 (1.19–1.73)	<0.001	1.28 (1.03–1.58)	0.026
≥ 1 year	1.44 (1.18–1.74)	<0.001	1.27 (1.02–1.58)	0.032
≥ 3 years	1.36 (1.08–1.70)	0.008	1.23 (1.08–1.46)	0.035

Abbreviations: OR, Odds ratio; CI, confidence interval; DM, diabetes mellitus

*The model was adjusted by sex, age, urbanization level, income, and comorbidities.

Table 4 lists the Odds ratios for acute epiglottitis with regards to anti-diabetic agents and diabetes related complications among diabetic patients in this study setting. Compared to the diabetic patients who did not receive anti-diabetic agents before the index date, the adjusted OR of epiglottitis was 1.02 (95% CI = 0.63–1.65. *p* = 0.929) for those who did take anti-diabetic agents before the index date. Use of anti-diabetic agents was not significantly associated with the development of acute epiglottitis among diabetic patients, including oral hypoglycemic agents (OHA) alone (aOR = 0.88, 95% CI = 0.53–1.46, *p* = 0.616), and OHA combined with insulin/ insulin alone (aOR = 1.30, 95% CI = 0.76–2.22, *p* = 0.339). The association between presence of diabetes complications and the occurrence of acute epiglottitis was also not significant among diabetic patients in this study setting (aOR = 0.86, 95% CI = 0.59–1.26, *p* = 0.439).

**Table 4:** Odds ratios for acute epiglottitis with regards to anti-diabetic agents and diabetes related complications among DM patients.

	Acute epiglottitis							
	Yes (n = 180)		No (n = 530)			Multivariate Model^†^		
Variable	n	%	n	%	*P*-value*	aOR	95% CI	*P*-value^a^
**Anti-diabetic Agents**					0.045			
No	31	17.2	89	16.8		1.00	(reference)	
Yes	149	82.8	441	83.2		1.02	(0.63–1.65)	0.929
OHA alone	76	42.2	276	52.1		0.88	(0.53–1.46)	0.616
Insulin^b^	73	40.6	165	31.1		1.30	(0.76–2.22)	0.339
**aDCSI score**					0.605			
aDCSI = 0	93	51.7	262	49.4		1.00	(reference)	
aDCSI≥1	87	48.3	268	50.6		0.86	(0.59–1.26)	0.439

Abbreviations: aOR, adjusted odds ratio; CI, confidence interval; OHA, oral hypoglycemic agents; DM, diabetes mellitus; aDCSI, adapted Diabetes Complications Severity Index

†Data were adjusted for sex, age, urbanization level, income, and comorbidities.

*p value of chi-squared test.

^*a*^P-value of multivariate logistic analyses

^*b*^ include OHA combined with insulin and insulin alone

## Discussion

To the best of our knowledge, this population-based case–control study is the first to elucidate the quantitative relationship between DM and acute epiglottitis. By using the nationwide population-based database, we overcame the difficulty of recruiting patients with a disease of low incidence and identified adequate numbers of epiglottitis cases with minimal selection bias, and this is because all health care services are covered by the NHI program in Taiwan. Based on the power of the large sample size, our study provides robust evidence for the higher odds ratio of underlying DM in patients with epiglottitis than in those without. To consider the effects of potential confounders, we used multivariate logistic regression after adjustment for comorbidities, including asthma, chronic liver disease, coronary artery disease, hypertension, and pneumonia/influenza, upper digestive tract cancer, autoimmune diseases, COPD, alcohol dependence and abuse, corrosive injury of upper digestive tract, and GERD, to compare the outcomes of the case and control groups. The association between DM and acute epiglottitis remained significant even after adjustment for a variety of comorbidities, and remained constant with different preexisting DM duration before the index date. Based on the results of this case control study, several comorbidities, including pneumonia and influenza, COPD, GERD, autoimmune diseases, and upper digestive tract cancer were also associated with the development of acute epiglottitis (Table 2). Therefore, it must be cautious in the interpretation of these results: although preexisting DM is a significant factor associated with the development of acute epiglottitis, other factors can play a role in contributing to the acute epiglottitis due to its multi-factorial characteristics. Subgroup analyses elucidated the significant associations between DM and acute epiglottitis in men and patients aged 35–64 years. By analyzing the use of anti-diabetic agents and aDCSI among diabetic patients in this study setting, we tried to correlate the severity of DM with occurrence of acute epiglottis. We found that among diabetic patients, taking anti-diabetic agents or not was not significantly associated with the development of acute epiglottitis. Similarly, patients with diabetes-related complications were not associated with increased occurrence of acute epiglottitis as compared to those without complication. These findings suggested the importance of blood glucose control and active management of diabetes complications regarding the occurrence of acute epiglottitis. In the future, prospective clinical trials are mandatory to elucidate the causal relationship between severity of DM and the development of acute epiglottitis.

Numerous studies have demonstrated epiglottitis to occur predominantly in men (54%–88%).[1, 15, 22, 30, 35, 36, 41, 42] In the present study, the subgroup analysis showed a significant association between DM and epiglottitis in men but not in women, supporting the male-predominant incidence in epiglottitis patients with DM as previously reported[23]; however, the underlying mechanism remains unclear. Studies revealed that men are more susceptible than women to most kinds of respiratory tract infections in adults and children[43].The role of androgens in the regulation of the immune system and disease resistance genes may contribute to the observed sex differences in the association between DM and epiglottitis[43–46]. Lifestyle, behavioral, and socioeconomic differences between men and women may also explain the observed findings[43].

The present study identified a significant association between DM and epiglottitis in patients aged 35–64 years, perhaps because patients in this age group tend to be relatively healthy with fewer comorbidities. Notably, a large number of patients with epiglottitis were younger than 34 years in this study (Table 2). However, patients with epiglottis in this age group had DM less frequently, and no statistical significance was observed. In patients aged older than 65 years, the weakened immune responses by aging[47] and increased underlying comorbidities may further attenuate the influence of DM on the risk of developing epiglottitis.

Studies have revealed that 3.5%–26.6% of patients with epiglottis have comorbid DM.[9, 12, 15, 23, 25, 27, 30] However, this association was never verified due to the disease rarity and the lack of control subjects for testing the corresponding statistical significance. In this study, we identified a significant association between DM and epiglottitis, which may be explained by the altered immunity from depressed polymorphonuclear leukocyte function[48–50]and decreased leukocyte adherence and phagocytic activity[51, 52] that make the patient more susceptible to acute epiglottitis. In addition, the most frequent respiratory tract infection associated with DM is caused by *S*. *pneumoniae*[53]—the most important bacterial etiology of acute epiglottitis in both adults[54] and vaccinated children.[19, 36] Therefore, we assume that the common respiratory infection in patients with DM and in epiglottitis shared the same pathogen, which may lead to the increased risk of epiglottitis among patients with DM and must be corroborated by future investigations. These findings suggest that underlying DM may play a role in the pathogenesis or pathophysiology of acute epiglottitis, which makes patients with DM more susceptible to acute epiglottitis.

Previous study indicated that the severity of epiglottitis is higher in DM patients due to the increased two days mortality and airway intervention rates.[11] A recent study reported that the outcome of critical epiglottitis patients was favorable if early respiratory tract protection could be adequately performed.[55] Therefore, identifying the risk factors for DM patients with epiglottitis who will probably require airway intervention is imperative in clinical decision-making to avoid fatal complications. Factors associated with an increased risk of airway obstruction in patients with epiglottitis include DM, stridor, muffled voice, hypoxia, drooling, rapid clinical course, a high pulse rate, and an epiglottic cyst or abscess.[9, 11, 21, 22, 30] Katori *et al*. analyzed 96 adult patients with epiglottitis and proposed that, under flexible laryngoscopy, severely swollen epiglottis and arytenoid/aryepiglottic folds with less than half of the posterior vocal folds visible were correlated with the requirement for airway intervention.[30] Flexible endoscopy enables early detection of acute epiglottitis and identifies the need for airway intervention. Those with precarious symptoms/signs and endoscopic findings should be observed intensively, or the airway should be secured immediately.[36] With appropriate and timely treatment, the prognosis of acute epiglottitis is usually favorable.[12, 15]

The major strength of this study is its large population size. To investigate the significance of underlying DM in the pathogenesis of acute epiglottitis, conducting a single-center study with a sufficient sample size and adequate follow-up time may not be feasible. The nationwide insurance claims database enabled us to investigate the risk factors for epiglottitis with a low selection bias. The statistical power and the precision of risk appraisal were also increased by the size of the study population. The NHIRD has been reported to be a valid source for population-based research with regular examinations of the accuracy of medical coding and the clinical records.[56] However, our study has limitations. First, innate information bias may exist because all diagnoses of the clinical conditions of interest and other comorbidities were based on ICD-9-CM codes in the claims records. Second, detailed information regarding the severity of DM, such as blood glucose levels, HbA1c levels, or dosages of anti-diabetic drugs, and epiglottitis related test/images were not available in the claims data. Therefore, the relationship between the severity and treatment outcomes of acute epiglottitis and the level of DM control could not be evaluated. Third, some suspected contributing risk factors for epiglottitis were unavailable from the insurance data, such as the personal history of alcohol and cigarette consumption[11, 12, 37]—a potential confounder that could not be adjusted for. Finally, we did not explore the underlying mechanism of the association between DM and acute epiglottitis. More research is warranted to validate our findings and to assess the association between the level of DM control and the occurrence and severity of acute epiglottitis.

In conclusion, the findings of this population-based case–control study suggest that DM is one of the possible factors associated with the development of acute epiglottitis. To achieve an early diagnosis and avert life-threatening complications of acute epiglottitis, physicians should always be aware of the symptoms/signs of acute epiglottitis in DM patients, particularly in men aged 35–64 years.
